# Whooping Cough

**Published:** 1876-08

**Authors:** 


Whooping-Cough.—Dr. Octavius Sturges, Assistant Physician to Hospital for Sick Children, Great Ormond St., London, presents (*Lancet*, May 20, 1876,) the following summary of his views concerning pertussis:

1. Whooping-cough is a nervous disease of immature life, due immediately, like nervous asthma, to a morbid exaltation of sensibility of the bronchial mucous membrane. Although possible in a modified form at all ages, it has its period of special liability and full development simultaneously with that time of life when the nervous system is irritable, and the mechanism of respiration diaphragmatic. A child of the proper age, with catarrh and cough, is thus on the very brink of whooping-cough. A large proportion of such children will develop the disease for themselves upon casual provocation, all contagion and all epidemic influence apart.

2. The whoop of whooping-cough is due to a spasmodic contraction of the diaphragm, which follows its extreme relaxation with the emptying of the lungs by spasmodic cough, the force of the inrush of air being met by a conservative spasm on the glottis.

3. The natural history and relations of whooping-cough—its uneven course, indeterminate duration, method of recovery and cure, frequent absence of pyrexia, and seasons of prevalence—are in striking contrast with diseases of the zymotic class. Admitting the fact of its contagion, the great commonness of the disease and its association with epidemic catarrh, coupled with the popular belief that its source of infection may be indefinitely remote, are circumstances which must combine to render whooping-cough more contagious in appearance than it is in fact.

4. In its character as a purely nervous disease whooping-cough may very well be contagious like other nervous affections of a quasi-voluntary kind. The assumption of a specific morbid poison is both hypothetical and gratuitous, or so nearly gratuitous that the facts it seems to explain are insufficient to counterbalance its inherent improbability.

5. The non-recurrence of whooping-cough is not, in strictness, analogous to the non-recurrence of contagious fevers, nor out of real harmony with the pattern of nervous disease. It is the rule that affections of this class alter their shape with the successive epochs of life, so that each will appear either solitary or recurrent, according as the time allotted for it is shorter or longer. The after-infancy period to which whooping-cough attaches is one of brief duration and special liabilities. The features of the disease are in strict correspondence with the characteristics of its time of life.

6. The specific remedies for whooping-cough (which have their season and may be said now to include all drugs, whatever, of any potency) have all of them a certain testimony in their favor. They agree in a single point; whether by their nauseousness, the grievous method of their application, or the disturbance they bring to the child's habits and surroundings, the best vaunted remedies—emetics, sponging of the larynx, ill-flavored inhalation, change of scene, beating with the rod—all are calculated to *impress* the patient, and find their use accordingly.—*Monthly Abstract of Medical Science.*

Camphorated Ether in Erysipelas.—According to Cavazzani, a preparation consisting of camphor and tannin, of each one part, and ether eight parts, is an excellent application, even in cases of phlegmonous erysipelas. It is also very efficacious in burns of the first and second degrees; the heat disappears rapidly, and blisters do not form. It should be applied every three hours.—*Medical Record.*

